# Survey of psychiatric assessment rooms in UK emergency departments

**DOI:** 10.1192/pb.bp.114.049742

**Published:** 2016-04

**Authors:** Jim Bolton, Lucy Palmer, Rohanna Cawdron

**Affiliations:** 1 St Helier Hospital, Surrey, UK; 2 College Centre for Quality Improvement, London, UK

## Abstract

**Aims and method** We aimed to estimate the proportion of UK emergency departments with a psychiatric assessment room and to determine whether such rooms met criteria for conducting high-risk assessments. Liaison psychiatry services were asked whether their hospital had such a room, whether it met the criteria and whether respondents judged it to be sufficiently safe and private.

**Results** Of the 60 emergency departments included in the survey, 23% had a psychiatric assessment room that met all the safety criteria and was judged to be safe and private. Barriers to the establishment of an appropriate facility included it being a low priority for hospital management, a room being used for other purposes, and balancing safety requirements with the creation of a calming environment.

**Clinical implications** Mental illness is a common reason for presentation to emergency departments. Despite national recommendations, this survey indicates that many departments lack a sufficiently safe and private assessment room, which compromises the safety and privacy of patient care.

Hospital emergency departments are involved with the resuscitation, assessment and treatment of patients with acute illness and injury. This includes patients with mental as well as physical illness. UK emergency departments see about 50 000 new patients each year, with a range of between 30 000 attendances for smaller departments and over 100 000 for the largest.^1^ The incidence of patients presenting with mental illness is about 5%.^1,2^ Common psychiatric presentations include self-harm, suicidal ideation, psychosis, and alcohol and substance misuse.^1,2^

Liaison psychiatry is the subspecialty of general psychiatry that deals with the management of mental illness in general medical settings.^3^ A multidisciplinary liaison psychiatry service covering in-patient wards and the emergency department is recommended as an essential requirement for all acute hospitals.^1,4,5^ It is also recommended that an emergency department should have an interview room for psychiatric consultations.^2,6,7^ Such a room should not be isolated from the main department and should be sufficiently safe and private. It should provide a calming environment and be equipped for assessments of patients whose mental illness increases their risk of harm towards themselves or others.^8^

The Psychiatric Liaison Accreditation Network (PLAN) is an initiative of the Royal College of Psychiatrists' Centre for Quality Improvement (CCQI) in partnership with the Royal College of Physicians, the Royal College of Nursing, the College of Emergency Medicine and the mental health charity Mind. Patient and carer representatives are integral to the setting of quality standards and accreditation of services. PLAN works with services to assure and improve the quality of psychiatric care in hospital settings. Liaison psychiatry services are audited against nearly 200 standards. These standards have been established following a literature review and by a consensus of the organisations involved in PLAN. The standards are revised regularly, in discussion with the services engaged in the accreditation process, following the emergence of new policy and in the light of their application.

To achieve accreditation a liaison psychiatry service that assesses patients in an emergency department must have access to an interview room that meets specific criteria for safely conducting ‘high-risk assessments’ (Box 1).^9,10^ The experience of PLAN has been that many liaison psychiatry services have struggled to achieve accreditation because of the lack of an assessment room, or because the room failed to meet one of the necessary criteria.

We aimed to survey liaison psychiatry services across the UK to estimate the proportion of emergency departments with a psychiatric assessment room and to determine whether such rooms met the PLAN requirements for conducting high-risk assessments.

## Method

The survey was conducted over 3 months between September and November 2013. A list of questions was circulated by email to UK liaison psychiatry services and given out to delegates at a national meeting of such services. We did not select services to take part in the survey. However, we aimed to achieve a wide circulation and relatively high response rate to ensure that we had information from emergency department across the UK, in hospitals of varying sizes, and serving rural, suburban and urban areas.

**Box 1** PLAN standards for a psychiatry interview room for conducting high-risk assessments

The following include the standards used in this survey.^9^ Subsequent revisions to the standards are given in bold.^10^

Facilities for high-risk assessments must ensure the safety of staff and service users. They should:

- be located close to or within the main emergency department or acute medical unit (revision: **be located within the main emergency department**);
- have a door which opens both ways and is not lockable from the inside (revision: **have at least one door which opens outwards and is not lockable from the inside**);
- have an observation panel or window;
- have a panic button or alarm system unless staff carry alarms at all times;
- only include furniture, fittings and equipment which are unlikely to be used to cause harm (revision: **for example sinks, sharp-edged furniture, lightweight chairs, tables, cables, televisions or anything else that could be used to cause harm or as a missile are not permitted**);
- ideally the room should include two doors (revision: **while not mandatory for accreditation, PLAN highly recommends that assessment facilities should have two doors to provide additional security; all new assessment rooms must be designed with two doors**);
- **not have any ligature points**.

Respondents were asked to identify the hospital in which they worked to ensure that no duplicate responses were included in the analysis. They were told that specific emergency departments would not be named in the publication of results.

Respondents were asked:

- whether their hospital had an emergency department (only those answering positively were included in the analysis);
- whether the emergency department had a psychiatric assessment room;
- where there was a room, whether it met each of the criteria for conducting high-risk assessments (Box 1);
- whether the respondent judged the room to be sufficiently safe and, if not, why not;
- whether the respondent judged the room to be sufficiently private and, if not, why not;
- other comments or suggestions regarding the use of, or PLAN criteria for, such rooms.

The responses were analysed to determine the proportion of emergency departments with a psychiatric assessment room and how many of these rooms met the PLAN criteria for safety and privacy. Common themes were identified in the free-text responses.

## Results

Responses were received from liaison psychiatry services working in 60 of the 245 emergency departments in the UK (response rate: 24%). Although we are not identifying individual services and hospitals in the published findings, we can confirm that we received responses from hospitals of a range of sizes and in various locations. Of these hospitals, 51 (85%) had a psychiatric assessment room within or close to the emergency department. Whether or not the psychiatric assessment rooms met the PLAN criteria for facilities and equipment is indicated in Table 1. Of the 51 rooms, 25 (49%) were judged by respondents to meet all of these criteria: 33 (65%) were judged as sufficiently safe and 46 (90%) as sufficiently private. Overall, 14 (23%) emergency departments had a room that met all the criteria and that was also judged to be sufficiently safe and private by liaison psychiatry staff. Common themes arising from the free-text responses are incorporated into the discussion.

**Table 1:** Number of rooms meeting safety criteria for facilities and equipment (*n* = 51)

Criterion	*n* (%)
Door opens both ways and is not lockable from inside	32 (63)

Observation panel or window	46 (90)

Panic button or alarm system	38 (75)

Furniture, fittings and equipment unlikely to cause harm	33 (65)

Two doors	38 (75)

## Discussion

Despite long-standing national recommendations, in this survey of UK liaison psychiatry services only 85% of emergency departments whose staff responded to the survey were reported as having a psychiatric assessment room. Of these rooms, 49% met all the safety criteria listed by PLAN. Overall, only 23% of emergency departments included in the survey had a psychiatric assessment room that met all the criteria for conducting high-risk assessments and was sufficiently safe and private.

This is the first such survey of psychiatric assessment rooms in emergency departments. Its findings concur with the perception of PLAN that many liaison psychiatry services are working in departments with inadequate facilities. The provision of an appropriate room is mandatory for a liaison psychiatry service to be accredited by PLAN. As well as meeting specific safety criteria for facilities and equipment, the room should also be judged to be safe and private by the majority of patients and peer reviewers involved in the accreditation assessment. Failure to meet mandatory standards is judged by PLAN to constitute ‘a significant threat to patient safety, rights or dignity’.^10^

A common reason for a room being judged to be unsafe by respondents was that it was not a dedicated facility for conducting psychiatric assessments and that it was used by emergency department staff for other purposes. One respondent noted that ‘the room is often filled with medical equipment (and) inappropriate furniture’. Another described how the room was also used as an overflow waiting area for patients. Although there is a need to use space efficiently in an emergency department, this has to be balanced against the need for safe and private facilities to manage the relatively high proportion of patients with mental illness. We are aware of psychiatric interview rooms which meet the PLAN criteria, but which are also used flexibly for the assessment of other patients and meetings with relatives and carers.

Another common issue raised by respondents was a room being too small, for example, ‘to allow space for a patient to move around if restless’. Although a minimum size for the room is not stipulated in the PLAN standards, whether the room is large enough to conduct safe assessments is judged by a visiting peer review team as part of the accreditation process. The National Institute for Health and Care Excellence (NICE) recommends that the room should be large enough to accommodate six people.^6^

Sufficient privacy to conduct psychiatric assessments is also assessed by peer reviewers. In the survey several respondents judged that the rooms in their emergency departments were not private. Most often this was because conversations in the room could easily be overheard by staff or patients outside, or because those in the room were too easily visible from outside.

Several respondents described the difficulties they faced in trying to secure a psychiatric assessment room in their local emergency department or in ensuring that the room was adequately equipped. A frequently cited problem was the perceived lack of influence with emergency department management. The implication in a number of responses was that the liaison psychiatry service was seen as being delivered separately from the emergency department and therefore having a low priority when considering the facilities provided. One respondent suggested that the provision of a psychiatric assessment room should be mandatory when a mental health service is commissioned.

Although liaison psychiatry services often work across the whole hospital, we support them being seen as integral to the work of the emergency department. PLAN encourages this by recommending that clinical staff and management from both liaison psychiatry and the emergency department meet regularly to discuss common issues. The survey identified examples of good working relationships where the liaison psychiatry services had successfully negotiated for the provision of an assessment room. One respondent expressed their thanks to the PLAN peer review team for their feedback on the inadequacy of the assessment room: ‘I used it at every opportunity until we got the money to rebuild’.

Several respondents raised the difficulties they had experienced in establishing a room that is equipped to meet safety requirements, but also provides a calming environment for an agitated or anxious patient. One room was described as being ‘similar to a cell [with] white stark walls’ and another as ‘poorly decorated, [with] no natural light and [as] not welcoming or comfortable’. We recognise the challenge in meeting safety standards and providing a calming environment in a busy emergency department. However, measures can be taken to achieve the latter, such as having a window which allows natural daylight, decorating in calming colours, and use of pictures without glass that are securely fixed to the walls.

The results of this survey, including the free-text comments and suggestions by respondents, have informed a revision of the PLAN standards for psychiatric assessment rooms.^10^ The changes have also brought the standards into line with those for assessment facilities for patients detained under Section 136 of the Mental Health Act 1983.^11^ The PLAN standards for a psychiatric assessment room have been included in guidance published by the College of Emergency Medicine.^7^ We hope that this will reinforce the importance of all emergency departments having such a facility and will give additional support to liaison psychiatry services and colleagues in an emergency department who are negotiating for the provision or equipping of a psychiatric assessment room.

## Limitations

This is not a survey of all emergency departments in the UK. Instead, we disseminated the survey to liaison psychiatry staff, who we thought would be best placed to judge the adequacy of facilities in their hospitals and to make judgements about the safety and privacy of assessment rooms. Therefore, the survey does not include emergency departments in hospitals where there is no liaison psychiatry service. In our survey, the proportion of UK emergency departments with an assessment room was 85%, but the figure for UK departments as a whole is unknown. We think it is likely that a hospital without a liaison psychiatry service is less likely to have a psychiatric assessment room; hence the overall proportion of departments in the UK with the recommended facilities is likely to be lower than that found in the survey.

There may be bias in those services that completed the survey, including a possibility that staff were more likely to respond if they were frustrated about a lack of appropriate facilities in their hospital.

Another limitation is the lack of an independent opinion of the appropriateness of facilities. An experience of PLAN is that external reviewers occasionally disagree with liaison psychiatry staff that the assessment room in their hospital meets the required standards. If this was the case in this survey, it would tend to overestimate the proportion of emergency departments with an adequate assessment room.

This survey audited rooms against the third edition of the PLAN standards, which have now been revised, taking account of the survey's findings.^9,10^ In view of the changes to some of the requirements the proportion of rooms meeting the standards is likely to have changed. However, we judge that any change would be relatively small and would not alter the overall conclusions of the survey.

## Implications

When psychiatric assessment rooms are being designed we strongly advocate that the PLAN and College of Emergency Medicine guidelines are followed to ensure provision of a sufficiently safe and private environment (Box 1). Although we are not aware of any specific planning guidance on the size of such a room, as previously noted, NICE guidance indicates that it should be large enough to hold six people. The design should also ensure that someone outside the room should have clear lines of sight for all areas via the observation panels or windows.

Despite recommendations that all emergency departments should have a psychiatric assessment room that is sufficiently safe and private, this survey indicates that such facilities are often lacking. Our findings concur with the experience of PLAN in its reviews of services and facilities. We believe that they are broadly representative of the current provision and equipping of psychiatric assessment facilities in UK emergency departments.

Mental illness is a common reason for presentation to emergency departments, where the absence of appropriate assessment facilities compromises the safety and privacy of patient care. Along with the College of Emergency Medicine and the other partners in PLAN we endorse the provision of psychiatric assessment rooms and urge inspectors of health services to address this in their own reviews of the quality and safety of patient care in emergency departments.
